# Bronchial Washing Fluid Versus Plasma and Bronchoscopy Biopsy Samples for Detecting Epidermal Growth Factor Receptor Mutation Status in Lung Cancer

**DOI:** 10.3389/fonc.2021.602402

**Published:** 2021-03-22

**Authors:** Xinyu Zhang, Chun Li, Maosong Ye, Qin Hu, Jie Hu, Ziying Gong, Jieyi Li, Xiaokai Zhao, Yiqing Xu, Daoyun Zhang, Yingyong Hou, Xin Zhang

**Affiliations:** ^1^Department of Respiratory Medicine, Zhongshan Hospital, Fudan University, Shanghai, China; ^2^Department of Pathology Medicine, Zhongshan Hospital, Fudan University, Shanghai, China; ^3^Department of R&D, Shanghai Yunying Medical Technology, Co. Ltd., Shanghai, China; ^4^Department of R&D, Jiaxing Yunying Medical Inspection Co., Ltd., Jiaxing, China; ^5^Division of Hematology and Oncology, Department of Internal Medicine, Maimonides Medical Center, Brooklyn, NY, United States

**Keywords:** lung cancer, bronchoscopy, plasma, bronchial washing fluid, EGFR

## Abstract

**Background:**

Bronchial washing fluid (BWF) is a common specimen collected during bronchoscopy and has been suggested to contain both tumor cells and cell-free DNA. However, there is no consensus on the feasibility of BWF in epidermal growth factor receptor (EGFR) genetic analysis because of the limited sample size and varying results in previous studies. This study compared the feasibility, sensitivity, and specificity of detecting EGFR mutation using BWF, bronchoscopy biopsy, and plasma samples in patients with lung cancer (LC).

**Materials and Methods:**

A total of 144 patients (110 with LC and 34 without LC) were enrolled in the study. During diagnostic bronchoscopy for suspected LC lesions, bronchial washing with saline was performed directly or through a guide sheath. BWF was collected as well as paired bronchoscopy biopsy and plasma samples, and EGFR mutation testing was performed *via* highly sensitive blocker polymerase chain reaction. The EGFR mutation status of histologic samples was set as the standard reference.

**Results:**

Compared with the histologic samples, the sensitivity, specificity, and concordance rate of EGFR mutation detected in BWF samples were 92.5%, 100%, and 97.9%, respectively. Moreover, BWF showed a higher sensitivity in EGFR mutation testing than both plasma (100% [8/8] vs. 62.5% [5/8], *p* = 0.095) and bronchoscopy biopsy samples (92.5% [37/40] vs. 77.5% [31/40], *p* = 0.012) and identified EGFR mutations in 6 cases whose biopsy failed to establish an LC diagnosis. The diameter of the target lesion and its contact degree with BWF were positive predictive factors for EGFR testing results.

**Conclusions:**

BWF yields a high sensitivity in EGFR mutation testing, having high concordance with histologic samples, and presenting the benefit of rapid EGFR mutation detection in LC patients.

## Introduction

Lung cancer (LC) is the most common cause of cancer-related mortality worldwide. Previous studies have proved that the epidermal growth factor receptor (EGFR) is a major molecule leading to the development of LC. Inhibition of EGFRs, such as tyrosine kinase inhibitors (TKIs), is confirmed to be clinically beneficial, especially in tumors that harbor active EGFR mutations (1). Several trials indicated that using TKIs instead of chemotherapy is a better choice for patients with EGFR mutant LCs, making EGFR a good therapeutic target for LC. Therefore, EGFR mutation testing is necessary and important for the initial diagnosis of LC (2).

Generally, tumor tissue samples are preferred for genetic analysis, but insufficient quantities of the sample is the main shortcoming across almost all populations (3). To guide treatment decisions in locally advanced and metastatic LC patients, many efforts have been made to rapidly detect EGFR mutations using less invasive methods, including the use of small biopsies, cytology specimens, and liquid biopsy samples (4, 5). However, testing of these samples also have limitations such as insufficient amounts, low accuracy, and long turn over periods. For example, circulating tumor DNA (ctDNA) specimens extracted from plasma samples may have low concentration, high fragmentation, and contamination of non-tumoral cell-free DNA (cfDNA) (6, 7). Although the specificity of detection in these specimens was near 100%, the sensitivity ranged from 67.5% to 75% (8–10).

Bronchial washing fluid (BWF) is the fluid recovered after washing the target pulmonary lesions using physiological saline during bronchoscopy. BWF may be a suitable specimen for molecular testing for the following reasons (1): compared with histology samples, fresh specimens have better DNA preservation; (2) compared with plasma samples, samples with direct correlation to tumor lesions may lead to the collection of larger amounts of tumor-related DNA; (3) the technique could be practical for patients with contraindications to biopsy procedures (bleeding or pneumothorax); and (4) the technique has the potential to reflect intratumoral heterogeneity (11, 12).

However, there is no consensus on the feasibility of BWF in EGFR genetic analysis because of the limited sample size and varying results in previous studies (13–15). Some studies performed EGFR mutation detection using cfDNA in BWF supernatant samples using the TaqMan Mutation Detection assay (sensitivity: approximately 0.5%) (16, 17). Although a high sensitivity of 88% had been observed in cases containing malignant or atypical cells, no EGFR mutation was found in cases without tumor cells in the pellet. As nearly half of clinical BWF samples are devoid of malignant cells (18), its clinical application is greatly limited until a more sensitive technique is applied. Ryu et al. reported genetic profiling of early-stage lung tumors using both supernatant and cytologic samples of BWF, but the conclusions were drawn from a small sample size and from resectable tumors only (19). Another study performed a similar test using extracellular vesicle-derived DNA isolated from BWF samples of LC patients (20); however, the extraction of exosomes is complicated and has limited popularity in modern clinical applications.

Based on our previous studies, we demonstrated that the detection rate of EGFR mutations in BWF samples was 100% concordant with that in histological samples, with an overall accuracy of 95.3% (21). In the present study, we aimed to further investigate the feasibility of BWF specimens in EGFR mutation testing using highly sensitive blocker polymerase chain reaction (PCR) (0.1%) and compare this with bronchoscopy biopsy and plasma samples. In addition, we attempted to clarify the impact of tumor lesion-related factors and bronchoscopic technical factors on EGFR-testing outcomes.

## Materials and Methods

### Ethics Approval

This study was conducted according to the principles of the World Medical Association Declaration of Helsinki and approved by the ethics committee of Zhongshan Hospital (IRB approval No. B2013-031). All participants provided written informed consent for this study.

### Patient Population and Study Design

Between October 2016 and April 2017, 144 patients with suspected LC, based on chest computed tomography (CT), who underwent diagnostic bronchoscopic biopsy were enrolled at Zhongshan Hospital, Fudan University (Shanghai, China). Exclusion criteria included cases with metastatic lung tumor. BWF and paired biopsy samples were prospectively collected from each patient. In patients not diagnosed with LC based on bronchoscopic biopsy samples, further sampling or clinical follow-up was undertaken. EGFR mutation detection was performed in all enrolled patients to evaluate the validity and reliability of using BWF samples.

### Bronchoscopy and Bronchial Washing

A fibrobronchoscope (BF-1TQ260; Olympus, Tokyo, Japan) was used for careful airway examination. For endobronchial visible lesions, endobronchial biopsy and bronchial washing were performed through the working channel of the wedged bronchoscope in the target segmental bronchus. For endobronchial invisible peripheral lesions, transbronchial lung biopsy was performed by experienced practitioners under the assistance of endobronchial ultrasound. The endoscopic ultrasonography (EBUS) model included an EBUS probe (20MHz mechanical-radial type, UM-S20-20R or UM-S20-17S; Olympus, Japan) and a guide sheath (GS) kit (K-203, Olympus, Japan).

While EBUS was being performed, the ultrasound probe and GS were inserted through the working channel of the bronchoscope into the target bronchus. After sonographic confirmation of the biopsy site, bronchoscopic forceps were advanced through the working channel on GS. Multiple forceps biopsies were performed through additional X-ray fluoroscopy assistance. Based on the sonographic view on the display, the location of the biopsy forceps was evaluated as within, adjacent to, or outside of the target lesion (22). The extent of the contact between the target lesion and the BWF was defined as the contact degree. Depending on the location of the biopsy forceps, the contact degree was classified as positive contact (PSC) and possible contact (PBC). We defined PSC as biopsies taken within the lesion and possible contact PBC as biopsies taken adjacent to the lesion or when invisible under EBUS. Instead of traditional bronchial washing, we administered 20–40 mL saline through the GS channel using a connected syringe and suctioned back the fluid into the syringe after 3–5 s. The volume of the recovered fluid was at least 6 mL. Time of washing, use of GS, recovery volume, and biopsy location in relation to the lesion were documented.

### Sample Collection and Pathological Diagnosis

At least 6 mL of BWF was collected from each patient and was centrifuged at 600 g for 10 min within 2 h of collection to separate the supernatant and sediment. Cell smears were randomly prepared using the sediments from 21 LC patients, and two independent pathologists performed tumor cell assessments. Biopsy samples were endorsed to the pathology laboratory for the formalin-fixed paraffin embedded (FFPE) procedure, routine staining, and pathological assessment. Plasma samples were obtained from 13 randomly selected LC patients and were stored in cfDNA blood collection tubes (Streck, NE, USA) until use.

### DNA Extraction and Genetic Analysis

EGFR analyses were performed immediately after collection in all prepared BWF specimens regardless of the pathologic diagnostic result of the donor. DNAPlus™ Human Circulating Nucleic Acid Kit (Yunying, Shanghai, China) was used to extract cfDNA from 1 mL of the supernatant samples and paired plasma samples. The extracted cfDNA was dissolved in 20 μL of Tris-EDTA buffer solution and was quantified using the Qubit 2.0 Fluorometer and Qubit dsDNA HS Assay kit (Thermo Fisher Scientific, MA, USA). Genomic DNA (gDNA) was extracted from both sediment and paired biopsy samples using the DNA FFPE Tissue Kit (Yunying, Shanghai, China).

EGFR gene detection was conducted on cfDNA and gDNA using the Alldetect™ EGFR Mutation Test Kit (Yunying, Shanghai, China), which is a modified detection method based on blocker PCR (sensitivity: 0.01–0.1%) (23). Within each reaction mixture, exon 28 was amplified as the internal control. The mutational status of a sample was determined by calculating the Δcycle threshold value (ΔCT value) between the mutant allele assay with the mutated probe and the gene reference assay with the wild-type probe. Samples with a ΔCT less than the cut-off CT value of 20 were designated as mutation positive. For histological samples, genomic DNA was extracted from FFPE blocks using the QIAamp DNA Mini Kit (Qiagen, Hilden, Germany), according to the manufacturer’s protocol. EGFR mutation status was detected *via* the amplified refractory mutation system (ARMS) PCR and was used as a standard reference.

### Statistical Analyses

Statistical analyses were performed using the SPSS software (SPSS Statistics 21.0; IBM, New York, United States). In LC patients, EGFR status assessment of histologic tumor samples was considered as the standard reference; in cases wherein the patients were diagnosed with benign diseases, we followed up for an adequate period and considered the confirmed outcomes as standard references. McNemar’s test was used to assess the significance of differences between BWF and histologic samples. The degree of consistency was assessed using the Kappa coefficient. The chi-square test or Fisher’s exact test was used for univariate analysis. Statistical significance was assessed using a two-tailed *p* value < 0.05.

## Results

### Patient Characteristics

**Figure 1:**
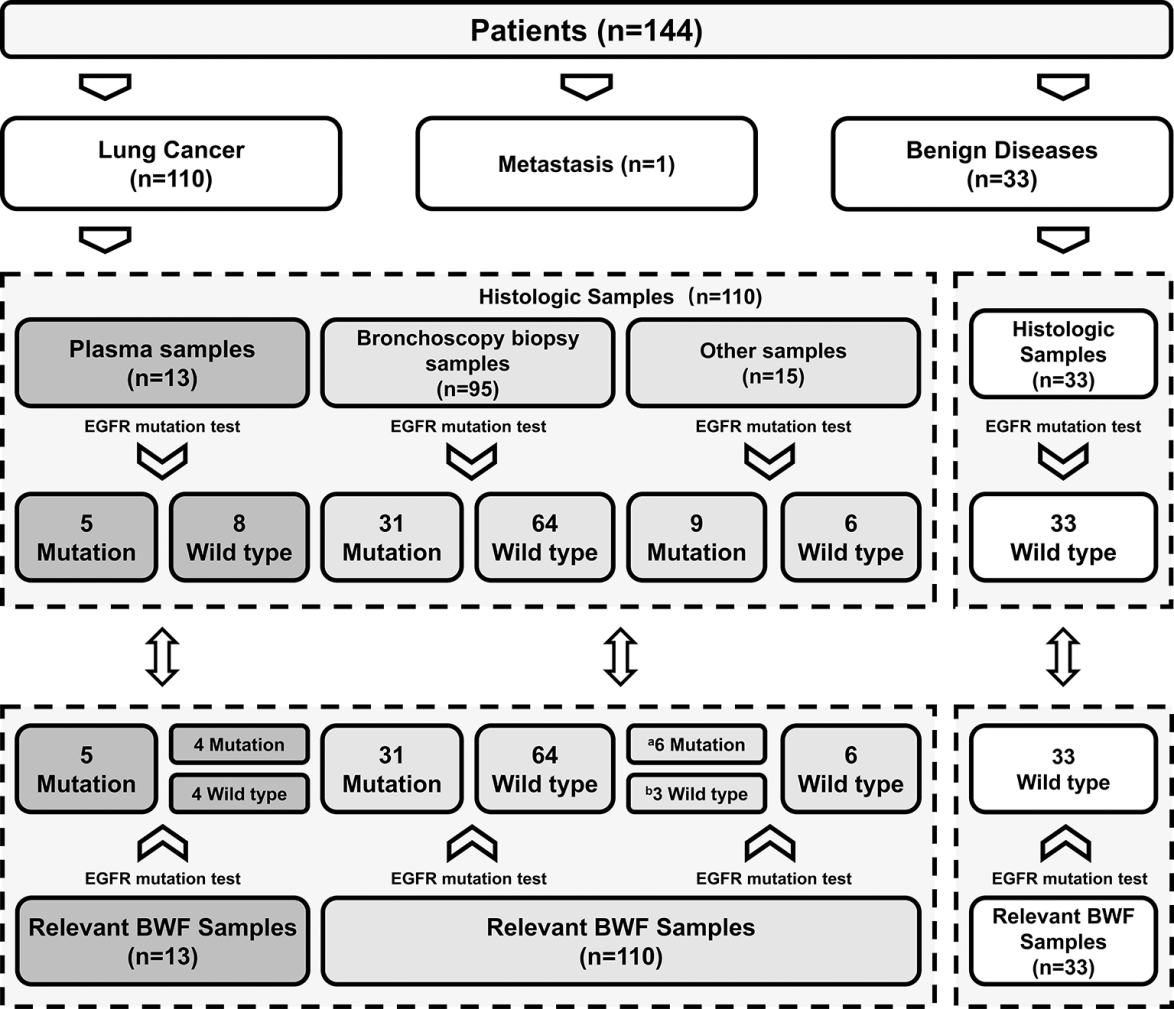
Comparison of EGFR mutation status between BWF and histologic samples. ^a^The cases with negative bronchoscopic biopsy results were detected as EGFR mutated type in BWF samples; ^b^False-negative cases. EGFR, epidermal growth factor receptor; BWF, bronchial washing fluid.

This study prospectively enrolled 144 consecutive patients with suspected LC. Primary lung malignancy was confirmed in 110 cases, whereas 33 were diagnosed with non-malignant diseases. One case of metastatic lung tumor was excluded. The clinicopathological characteristics of the patients are listed in **Table 1**. Of the 110 LC cases, 95 were diagnosed through bronchoscopic biopsy, with a diagnostic accuracy of 86.4% (95/110). In the 15 remaining patients whose biopsy results were negative, histologic and molecular diagnoses were based on tumor samples obtained using other sampling methods (7 cases of surgical resection, 3 of fine-needle aspiration, 2 of CT-guided percutaneous core needle biopsy, and 1 of transbronchial needle aspiration) or using clinical-radiological surveillance (2 cases) (**Figure 1**).

**Table 1:** Patient characteristics.

	Diagnosed with lung cancer (n = 110)	Not diagnosed with lung cancer (n = 33)
Demography, n (%)		
Male	64 (58.2%)	22 (66.7%)
Median age, yrs (range)	62.9 (29–87)	59.2 (35–79)
Never-smoker	69 (62.7%)	19 (57.6%)
Bronchoscopy modalities, n (%)		
EBB	40 (36.4%)	4 (12.1%)
TBLB	70 (63.6%)	29 (87.9%)
Histologic type, n (%)		
ADC	61 (55.5%)	
SCC	31 (28.2%)	
SCLC	10 (9.1%)	
LCNEC	2 (1.8%)	
Large cell carcinoma	2 (1.8%)	
Adeno-squamous carcinoma	1 (0.9%)	
^a^Others	3 (2.7%)	
EGFR mutated subtype, n (%)		
Exon 19 (deletion)	10 (9.1%)	
Exon 21 (L858R)	27 (24.5%)	
Exon 21 (L861Q)	2 (1.8%)	
Exon 20 (Insertion)	1 (0.9%)	
Wild type	70 (63.6%)	33 (100%)

a including one with NSCLC-NOS, and two clinically diagnosed with LC.

EGFR, epidermal growth factor receptor; EBB, endobronchial biopsy; TBLB, transbronchial lung biopsy; ADC, adenocarcinoma; SCC, squamous cell carcinoma; SCLC, small cell lung cancer; LCNEC, large cell neuroendocrine carcinoma; NSCLC-NOS, non-small cell lung cancer-not otherwise specific; LC, lung cancer.

### Factors Related to Better Outcomes

**Figure 2:**
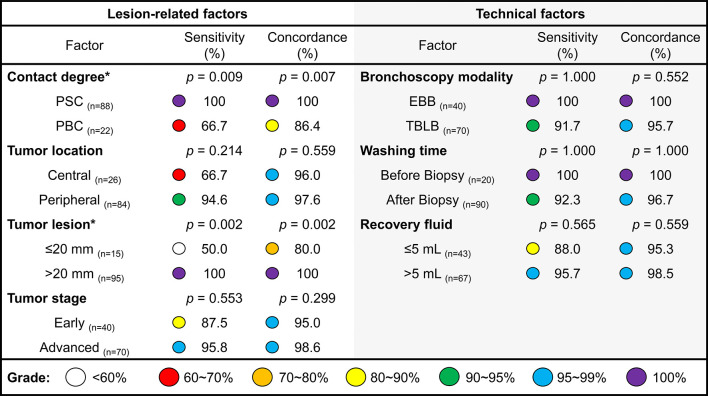
Influencing factors of using BWF samples in EGFR testing. The lesion-related and technical factors in BWF collection. The colored dots indicate the different grade of sensitivity or concordance ratios in EGFR testing results. PSC, positive contact; PBC, possible contact; EBB, endobronchial biopsy; TBLB, transbronchial lung biopsy.

Using BWF samples, we first explored the clinical factors related to better EGFR test results (**Figure 2**). In patients whose biopsies were evaluated as central to the lesion, BWF samples successfully detected all 31 cases with EGFR mutation, with the concordance rate being significantly better than that of biopsies taken from the periphery of the lesion (sensitivity: 100% vs. 66.7%, *p* = 0.009; concordance rate: 100% vs. 86.4%, *p* = 0.007). In addition, larger tumor diameters (≥20 mm) showed a trend toward higher sensitivity (100% vs. 50%, *p* = 0.002) and concordance rate (100% vs. 80%, *p* = 0.002). Other factors, including tumor location, tumor stage, bronchoscopy modalities, time of washing, and recovery volume were not significant.

### Comparison of EGFR Genotyping Results Between BWF and Plasma Samples

**Figure 3:**
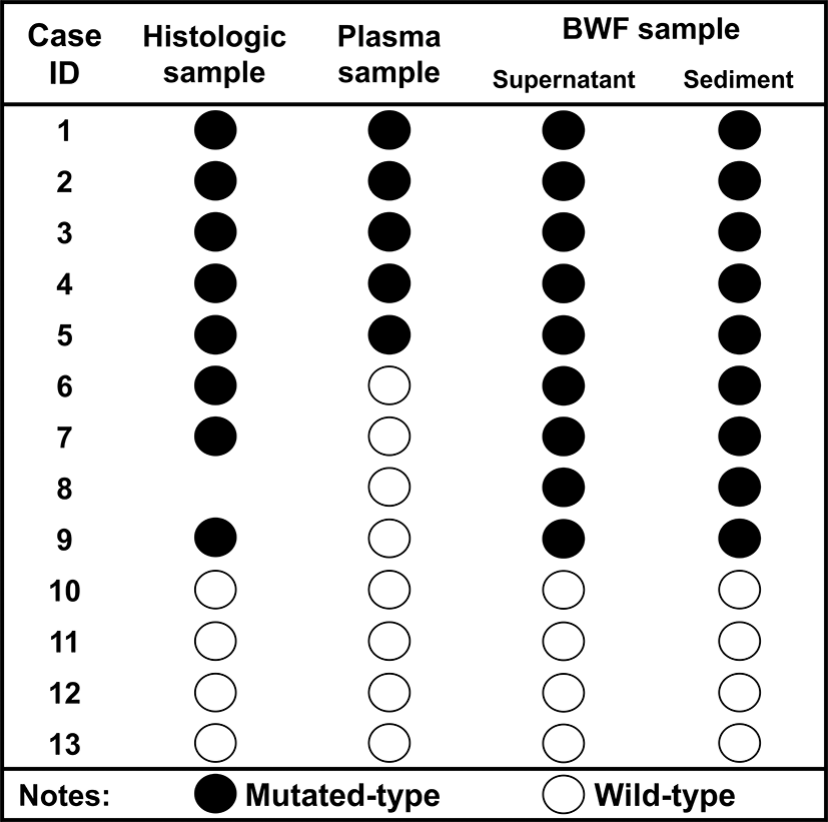
Comparison between BWF and plasma samples in EGFR testing. Black dots indicate the EGFR mutation type, and white dots indicate the EGFR wild type.. EGFR, epidermal growth factor receptor; BWF, bronchial washing fluid.

The Alldetect™ kit was used to perform the comparison of EGFR mutation detection in the pairwise BWF and plasma samples. The plasma samples were obtained at the same time as BWF samples (within one day). A total of 13 BWF and plasma sample pairs from LC patients were randomly included in the analysis, of which 8 were confirmed to be EGFR mutated-type paired histologic samples and 4 were wild-type (**Supplementary Table S1**). BWF samples, both supernatant and sediment, had 100% (8/8) concordant results with histologic samples. By contrast, plasma samples failed to detect 3 cases harboring EGFR mutation, with a sensitivity of 62.5% (5/8) and concordance rate of 75% (9/12) (**Figure 3** and **Supplementary Table S1**). Notably, one case was excluded since no histologic specimens were obtained (case 8 in **Figure 3** and **Supplementary Table S1**).

### Comparison of EGFR Genotyping Results Between BWF and Bronchoscopy Biopsy Samples

We successfully performed EGFR mutation detection in all 143 cases. Using BWF samples, the average duration of the bronchoscopic procedures for EGFR mutant results was 2 days, which was significantly shorter than the 11.8 days average duration when histologic samples are used (range: 1–36 days; median, 10.5 days) (*p* < 0.001). In all 33 non-malignant cases, EGFR mutation status was identified as wild-type, using both histologic and BWF samples. Among the 95 LC patients with bronchoscopy biopsy samples, EGFR mutations were identified in 31 patients. Paired BWF samples identified the same mutation in all these cases, with a sensitivity of 100% and specificity of 100%. Out of the 15 LC patients who failed to be diagnosed through the initial bronchoscopic biopsy, 9 patients were found to harbor EGFR mutations in the BWF samples. These mutations were then identified through further biopsy sampling (N=8) or clinical follow-up (Case 6, which was depicted in case representation) (**Table 2**). BWF samples still revealed the presence of EGFR mutations in 6 cases despite the negative biopsy results (**Figure 1**). Overall, BWF achieved a sensitivity of 92.5% (37/40), specificity of 100% (103/103), a positive predictive value of 100% (37/37), and a negative predictive value of 97.2% (103/106). The overall concordance rate between BWF and histologic samples was 97.9% (140/143) (**Supplementary Table S2**). Interestingly, BWF yielded a higher sensitivity in EGFR testing than the bronchoscopic biopsy samples (92.5% [37/40] vs. 77.5% [31/40], *p* =0.012).

**Table 2:** Detailed information of LC patients harboring EGFR mutation with negative results at initial biopsy.

Case	Contact degree	Lesion size (mm)	Clinical diagnosis	EGFR mutation		
				Histologic samples (sampling method)	BWF samples	Follow-up BB samples
1	PSC	45*30	ADC	L858R (Surgery)	L858R	L858R
2	PSC	25*17	ADC	L858R (Surgery)	L858R	L858R
3	PSC	46*30	ADC	E19Del (FNA)	E19Del	E19Del
^*^4	PSC	46*30	LC	L858R (Plasma)	L858R	WT
^*^5	PBC	26*17	LC	L858R (Plasma)	L858R	WT
^†^6	PSC	42*22	ADC	WT (Surgery)	L861Q	WT
7	PBC	20*13	ADC	L858R (TBNA)	WT	WT
8	PBC	21*20	ADC	L858R (FNA)	WT	WT
9	PBC	14*10	ADC	L858R (Surgery)	WT	WT

^*^Clinically diagnosed with LC, EGFR-L858R mutation was detected both in BWF and plasma. The tumor showed partial response (PR) to EGFR-TKIs. ^†^Case represented.

EGFR, epidermal growth factor receptor; EGFR-TKIs, epidermal growth factor receptor tyrosine kinase inhibitors; BWF, Bronchial washing fluid; BB, bronchoscopic biopsy; PSC, positive contact; PBC, possible contact; ADC, adenocarcinoma; LC, lung cancer; FNA, fine-needle aspiration; TBNA, transbronchial needle aspiration; WT, wild type.

Notably, the concordance between the BWF supernatant and BWF sediment was 100%, even though nearly half of the BWF sediment samples were observed to have ≤ 10% tumor cells in the 21 randomly selected cases (12/21, **Supplementary Table S3**). EGFR mutations were detected in 6 cases, of which 2 cases had absence of tumor cells.

### Case Representation

**Figure 4:**
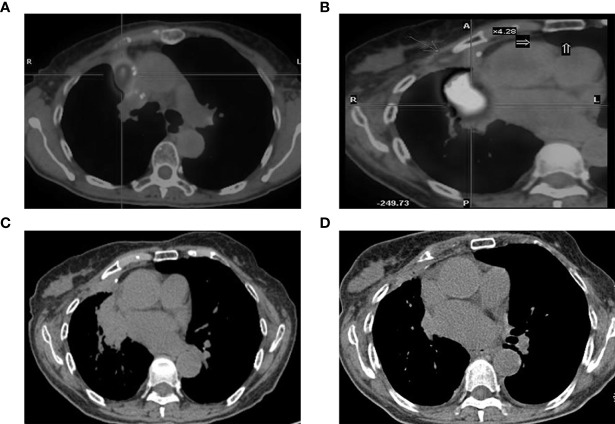
CT findings. **(A)** tumor status at diagnosis; **(B)** the increased tumor progress; **(C)** chest CT scan result before the afatinib treatments and **(D)** the chest CT scan result after two months of afatinib treatments. CT, computed tomography.

There was one patient whose EGFR mutation status was identified only through BWF samples (Case 6 in **Table 2**). According to the record, the patient had adenocarcinoma, which was treated surgically in 2008. Four years later, the tumor relapsed as a right hilar mass and was identified as EGFR wild-type through histological samples; the patient then received radiotherapy and chemotherapy (**Figure 4**). In May 2017, the tumor progressed with an increased right pulmonary mass (**Figure 4**). Bronchoscopic biopsy failed to obtain adequate tumor tissue to establish the diagnosis, and EGFR mutation detection using forceps biopsy and plasma samples showed EGFR wild-type. However, in both supernatant and sediment BWF samples, EGFR mutations in exon 21 (L861Q) were detected. The patient received afatinib for two months and showed an improvement in clinical status. A chest CT revealed good partial response (**Figures 4C**). The patient has remained stable to date, i.e., 30 months after the initial diagnosis. This case suggests that BWF could be a reliable basis to determine EGFR mutation status in biopsy-negative cases, avoiding further invasive biopsy procedures.

## Discussion

Following our previous report (21), the present study further demonstrated that BWF samples can provide sufficient material in either the cell-free supernatant or the cell pellet for accurate genetic analysis of EGFR mutation status. Upon review of the epidemiological data in the Asian population, 40-55% of patients with non-small cell lung cancer were found to harbor EGFR mutations. E19Del (40-49%) and L858R (39-47%) are the most common subtypes of EGFR mutations, and the remaining mutations such as E20Ins (2.3-4.5%) and L861Q (1.3-1.9%) are uncommon (24). However, due to the small sample size, our results showed a certain deviation from these epidemiological data: In our study, EGFR mutations were detected in 36.4% of patients in the lung cancer cohort (40/110). Among them, 25% (10/40) had the E19Del mutation, 67.5% (27/40) had the L858R mutation, 5% (2/40) had the L861Q mutation, and 2.5% (1/40) had the E20Ins mutation. Further, compared with analysis results from tumor histologic samples, the sensitivity of BWF in EGFR mutation detection was 92.5% and the concordance rate was 97.9%. Based on these results, BWF appears to be an excellent source for EGFR mutation analysis.

Our results have potential clinical significance in comparing BWF with other sample sources for EGFR detection. Although liquid biopsy using cfDNA extracted from plasma is non-invasive, BWF has several advantages over plasma samples. First, the cfDNA obtained in the serum/plasma is low in concentration (8–60 ng/mL), with the quantity varying with tumor burden (25–27). By contrast, BWF has the advantage of yielding a much higher level of cfDNA concentration (58.21 ng/μL), according to a previous report (16). Presumably, there is direct shedding of tumor-related material into the BWF. Second, with the plasma specimen, even with highly sensitive droplet digital PCR and next-generation sequencing (NGS), the sensitivity of detecting EGFR-sensitive mutations in advanced or recurrent non-small cell LC patients has plateaued at around 76–79% (28–31) and is even lower (33%) in early-stage patients (32). Using the Alldetect™ kit, BWF demonstrated a higher sensitivity than paired plasma (100% vs. 62.5%) (**Supplementary Table S1**). Therefore, BWF may be potentially superior to plasma as a sampling source for genetic analysis.

Moreover, our study showed that BWF has a detection accuracy comparable with that of the tissue specimen in patients whose bronchoscopic biopsy could obtain adequate tumor tissue for pathology diagnosis. Therefore, BWF can be considered as a backup or a substitute source, depending on the clinical setting. For peripheral lesions, the overall diagnostic yield of bronchoscopic biopsy is 58–77%, despite the assistance of the ancillary technique (33). In patients whose bronchoscopic biopsy is not successful in establishing the diagnosis of malignancy, BWF could still identify patients harboring EGFR mutations, which can be an alarm to trigger further workup. Its suboptimal sensitivity in this setting likely is because of scant shedding from the tumor lesion owing to the lesion’s small size or peripheral location. In addition, BWF samples also allowed immediate detection and shortened the waiting period to access to therapeutic-instructive EGFR results to 1–2 days, which is significantly shorter than that of histologic samples (average 11.8 days, *p* < 0.001).

**Figure 5:**
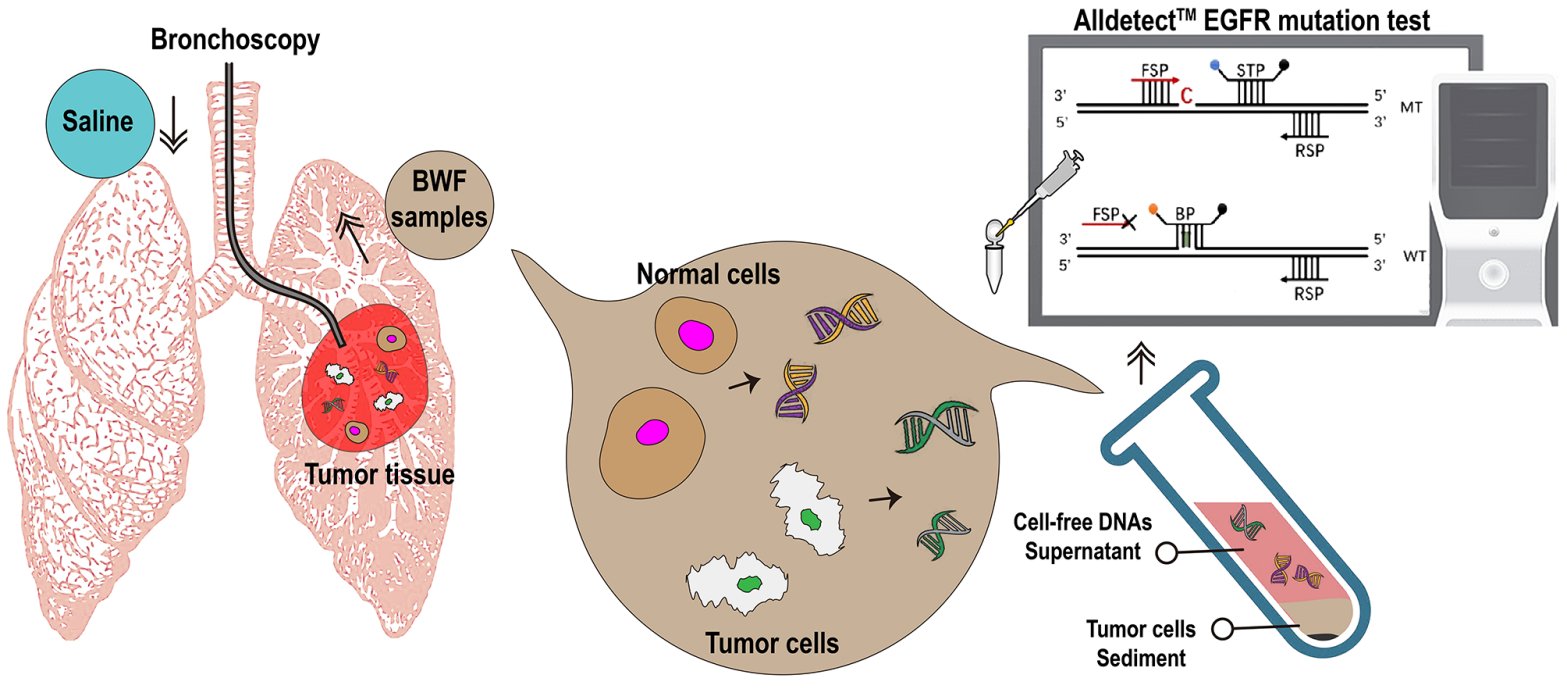
Summary diagram. EGFR, epidermal growth factor receptor; BWF, bronchial washing fluid; FSP, forward specific primer; STP, specific TaqMan probe; BP, block probe; RSP, reverse specific primer; C, cytosine; MT, mutation type; WT, wild type.

We also attempted to establish the criteria for BWF collection and promote its routine application. A small amount of saline (20 mL) could guarantee a mean recovery volume of 5.7 mL (range: 1.5–15 mL) in our study, making bronchial washing a safe and less invasive procedure. Only 1 mL of the recovery fluid could already meet the needs for gene detection, using highly sensitive blocker PCR. The presence of tumor cells, as confirmed on cytologic examination, does not seem obligatory for a successful detection using BWF supernatant cfDNA, and similar results were reported for cell paucity or cytologic-negative malignant pleural fluid specimens (34). We hypothesised that the origins of cfDNA in the BWF supernatant may derive from apoptotic and necrotic tumor cells or active release of living tumor cells (**Figure 5**). Further investigations in larger cohorts are expected to improve the criteria for the clinical application of BWF sampling.

With the high yield of cfDNA found in BWF, further development for expansion of testing from NGS to other genomic markers, such as ALK, KRAS, PIK3CA, ROS1, BRAF, ERBB2, and many others, as well as immune therapy-related markers, is conceivable and promising. In addition, BWF collected through bronchoscopy is a relatively non-invasive source compared with bronchoscopic biopsy. Future directions could be studies on BWF without concurrent biopsy, and if similar success can be achieved, then BWF could be an optimal method of sample collection in cases with high risk of complications for bronchoscopic biopsy. Our results illustrated the potential superiority of BWF samples over plasma samples in EGFR mutational analysis; however, these results need further verification in larger cohorts.

## Conclusion

In conclusion, BWF is a reliable and sensitive specimen for the evaluation of EGFR mutation status, yielding a higher sensitivity and concordance with histologic samples and a shorter waiting time. As a parallel or alternative source to bronchoscopic biopsy samples, BWF has potential in the rapid detection of EGFR mutations and other gene alterations in LC patients.

## Data Availability Statement

The raw data supporting the conclusions of this article will be made available by the authors, without undue reservation.

## Ethics Statement

The studies involving human participants were reviewed and approved by Ethics committee of Zhongshan Hospital. The patients/participants provided their written informed consent to participate in this study.

## Author Contributions

XYZ, CL, MY, DZ, ZG, YX, YH, and XZ participated in the design and coordination of the study, provided the study materials, and helped to review the manuscript. XYZ, CL, QH, and JH performed the experiments. XYZ, JL, and XKZ analyzed the data and contributed to writing and editing the manuscript. JL performed data visualization. All authors contributed to the article and approved the submitted version.

## Funding

This research was supported by a grant provided by the Shanghai Three-Year Development Plan Project (15GWZK0102), the Shanghai Science and Technology SMEs Technology Innovation Fund (1702H117500), the Jiaxing Leading Talent Entrepreneurship Project; and the Technology innovation projects of Jiaxing.

## Conflict of Interest

Authors ZG, JL, XkZ, and DZ are employed by Shanghai Yunying Medical Technology, Co. Ltd. and by Jiaxing Yunying Medical Inspection Co. Ltd.

The remaining authors declare that the research was conducted in the absence of any commercial or financial relationships that could be construed as a potential conflict of interest.
