# Supplementary material for: Bronchial Washing Fluid Versus Plasma and Bronchoscopy Biopsy Samples for Detecting Epidermal Growth Factor Receptor Mutation Status in Lung Cancer
Source: Front Oncol. 2021 Mar 22;11:602402. doi: 10.3389/fonc.2021.602402 (PMC8020887; doi:10.3389/fonc.2021.602402)
Supplement: Supplementary file 1 [file Table_1.docx]

**Supplementary Table S1.** Comparison of EGFR mutation detection in BWF and paired plasma samples

| ID | Pathologic diagnosis | Tumor Stage | Histologic sample | Plasma sample | BWF sample | |
| --- | --- | --- | --- | --- | --- | --- |
|  |  |  |  |  | Supernatant | Sediment |
| 1 | ADC | Advanced | E19Del | E19Del | E19Del | E19Del |
| 2 | ADC | Advanced | E19Del | E19Del | E19Del | E19Del |
| 3 | ADC | Advanced | L858R | L858R | L858R | L858R |
| 4 | ADC | Advanced | E20Ins | E20Ins | E20Ins | E20Ins |
| 5 | SCC | Early | L858R | L858R | L858R | L858R |
| 6 | ADC | Advanced | E19Del | WT | E19Del | E19Del |
| 7 | ADC | Advanced | L858R | WT | L858R | L858R |
| 8 | ADC | Advanced | / | WT | L861Q | L861Q |
| 9 | ADC | Early | L858R | WT | L858R | L858R |
| 10 | ADC | Advanced | WT | WT | WT | WT |
| 11 | LCNEC | Advanced | WT | WT | WT | WT |
| 12 | SCC | Early | WT | WT | WT | WT |
| 13 | SCC | Early | WT | WT | WT | WT |
| Statistics | | Sensitivity | | 62.5 % | 100 % | 100 % |
|  |  | Specificity | | 100 % | 100 % | 100 % |
|  |  | Concordance rate | | 75 % | 100 % | 100 % |

**Notes:**

(1). BWF samples were detected by Alldetect^TM^ kit.

(2). Histologic sample was used as the control group.

(3). The statistical analyses exclude case 8 as there is no control histologic sample.

(4). *Abbr.* EGFR, epidermal growth factor receptor; BWF, Bronchial washing fluid; ADC, adenocarcinoma; SCC, squamous cell carcinoma; LCNEC, large cell neuroendocrine carcinoma; WT, wild type
