# Supplementary material for: Bronchial Washing Fluid Versus Plasma and Bronchoscopy Biopsy Samples for Detecting Epidermal Growth Factor Receptor Mutation Status in Lung Cancer
Source: Front Oncol. 2021 Mar 22;11:602402. doi: 10.3389/fonc.2021.602402 (PMC8020887; doi:10.3389/fonc.2021.602402)
Supplement: Supplementary file 2 [file Table_2.docx]

**Supplementary Table S2.** EGFR mutation Status Summary, Concordance, Sensitivity, Specificity, and Positive- and Negative-Predictive Value for Histologic vs. BWF Samples by EGFR Mutation Status (n=110)

|  | BWF EGFR Mutation Status (n) | | **Total** |
| --- | --- | --- | --- |
|  | **M+** | **M-** |  |
| Histologic EGFR mutation status, *n* |  |  |  |
| M+ | 37 | 3 | 40 |
| M- | 0 | 103 | 103 |
| Total | 37 | 106 | 143 |
| Sensitivity=92.5% (37 BWF M+ out of 40 histologic M+) | | | |
| Specificity=100% (all 103 histologic M- were BWF M-) | | | |
| Positive Predictive Value=100% (all 37 BWF M+ were histologic M+) | | | |
| Negative Predictive Value=97.2% (103 histologic M- out of 106 BWF M-) | | | |
| Concordance=97.9% (BWF and histologic results agreed in 140 of 143 cases)^a^ | | | |

**Notes：**

(1). ^a^ *P value* = 0.250 (>0.05); Kappa coefficient 0.95.
(2). *Abbr.* EGFR, epidermal growth factor receptor; BWF, Bronchial washing fluid; M+, mutation positive; M-, mutation negative
