# Supplementary material for: Bronchial Washing Fluid Versus Plasma and Bronchoscopy Biopsy Samples for Detecting Epidermal Growth Factor Receptor Mutation Status in Lung Cancer
Source: Front Oncol. 2021 Mar 22;11:602402. doi: 10.3389/fonc.2021.602402 (PMC8020887; doi:10.3389/fonc.2021.602402)
Supplement: Supplementary file 3 [file Table_3.docx]

**Supplementary Table S3.** The number of tumor cells in the sediment of BWF

| Case | Pathologic diagnosis | Washing time  (before/after forceps biopsy) | Tumor cell numbers | Percentage of tumor cells (%) | EGFR mutation |
| --- | --- | --- | --- | --- | --- |
| 1 | ADC | After | 0 | 0 | E19Del |
| 2 | ADC | After | 0 | 0 | L858R |
| 3 | ADC | After | 11 | 1-10 | L858R |
| 4 | ADC | After | 50-100 | 1-10 | E19Del |
| 5 | ADC | After | 100-300 | 90-100 | L858R |
| 6 | ADC | After | 100-300 | 1-10 | L858R |
| 7 | ADC | After | 0 | 0 | WT |
| 8 | ADC | After | 10-30 | 50-100 | WT |
| 9 | ADC | After | 100-300 | 1-10 | WT |
| 10 | ADC | After | 100-300 | 50-100 | WT |
| 11 | ADC | After | 300-500 | 50-100 | WT |
| 12 | ADC | After | 300-500 | 80-100 | WT |
| 13 | LUSC | Before | 0 | 0 | WT |
| 14 | LUSC | After | 0 | 0 | WT |
| 15 | LUSC | After | 0-10 | <1 | WT |
| 16 | LUSC | After | 100-300 | 1-10 | WT |
| 17 | LUSC | After | 100-300 | 10-30 | WT |
| 18 | SCLC | After | 100-300 | 50-100 | WT |
| 19 | SCLC | Before | 10-30 | <1% | WT |
| 20 | SCLC | Before | 11 | 10-30 | WT |
| 21 | NSCLC-nos | After | 50-100 | 50-100 | WT |

**Note：**

*Abbr.* EGFR, epidermal growth factor receptor; BWF, Bronchial washing fluid; ADC, adenocarcinoma; LUSC, lung squamous cell carcinoma; SCLC, small cell lung cancer; NSCLC-nos, non-small cell lung cancer, not otherwise specific; WT, wild type
